# The Relationship Between Food Environments and Health Outcomes: A Case Study in Lansing, Michigan

**DOI:** 10.3390/ijerph22101589

**Published:** 2025-10-20

**Authors:** Zeenat Kotval-K, Olivia R Nedd

**Affiliations:** Urban & Regional Planning Program, School of Planning, Design & Construction, Michigan State University, 552 W, East Lansing, MI 48824, USA

**Keywords:** food environments, health, fast-food outlets, convenience stores, food insecurity

## Abstract

Chronic disease, for which diet is a major risk factor, remains the leading cause of death in the United States, responsible for 8 out of 10 deaths. A continually growing body of research has been looking at food environments, relating them to characteristics of residents living in those environments and impacts on health outcomes. However, most of the research has been looking at Body Mass Index or obesity as the primary health outcome of such environments. This study looks at multiple health outcomes (chronic and perceived) from the Center for Disease Control’s PLACES—Local Data for Better Health dataset for Lansing, Michigan, and assesses the corresponding food environments, specifically the prevalence of fast-food outlets and convenience stores, to assess the impacts these food environments have, either directly or indirectly, on health. We find that fast-food outlets have a direct impact on certain health outcomes, while convenience stores impact certain health outcomes indirectly through food insecurity. These findings suggest that strategically balancing such environments with healthier options in underserved areas could help improve overall health.

## 1. Introduction

Preventable chronic diseases, for which diet is a major risk factor, are the leading cause of death and disability in the United States [1]. There exists a continually growing body of research examining the characteristics of the food environment, whether it represents areas with low availability of healthy and nutritious produce known as ‘food deserts’ [2,3] or areas with a high prevalence of unhealthy or fast-food places known as ‘food swamps’ [4].

To understand the impacts of the food environment on health, many studies use obesity or Body Mass Index (BMI) as a proxy for negative health outcomes, relying on the dominant chain of causality where obesity is a risk factor for some chronic diseases. However, an over-reliance on obesity as a mediator may be unreliable due to the subjectivity of the measurement and the fact that many chronic diseases, while being diet-related, may not be mediated by obesity.

If the nature of an individual’s food environment does correlate strongly with health outcomes, this would suggest that policy and other interventions meant to address disparities in food environments may actually lead to substantial changes in health outcomes, especially among disadvantaged communities, who tend to live in unsupportive food environments (e.g., few supermarkets, limited availability of fruits and vegetables, high availability of high-calorie foods and sugar-sweetened beverages). Given that resources and funds for interventions are limited and that even the most well-meaning interventions and policies can backfire, it is important to grasp the nature and strength of the relationship before solutions are applied.

This study aims to assess the relationship between food environments, specifically fast-food outlets and convenience stores, and health outcomes (other than just BMI/obesity) in the capital city of Lansing, Michigan.

### 1.1. Food Environment Characterizations

Disproportionately, racial minorities live in economically disadvantaged neighborhoods where access to grocery stores selling fresh produce is limited and unhealthy food stores such as fast-food chain stores are increasingly prevalent [5,6]. This discrepancy gets magnified in rural areas due to the lack of availability of healthy and nutritious produce near resident homes in general [7]. Similarly, lower-income neighborhoods have been shown to have fewer supermarkets and more stores selling foods with lower nutrition values [6,8]. This disproportionate burden of unhealthy food environments is also faced by renters and single-parent households [9].

Food swamps, areas with a high prevalence of convenience stores and fast-food outlets, have been known to correlate with increased dietary intake of unhealthy foods [2,10]. In essence, research has shown that there are racial and economic inequities when it comes to the prevalence of food deserts or food swamps [11,12,13]. Additionally, neighborhood food environments have a direct effect on dietary intake, where a higher prevalence of supermarkets is correlated with higher consumption of fresh fruits and vegetables and a higher prevalence of convenience stores is correlated with lower consumption of fresh fruits and vegetables [14].

### 1.2. Food Insecurity and Health Outcomes

When discussing vulnerable and underserved population groups and their food environments, food insecurity forms a natural connection. Food insecurity refers to a condition when households or residents lack access to adequate food due to limited resources (economic or otherwise) [15]. Vulnerable population groups facing food insecurity have been shown to have a poor diet, eat more at fast-food outlets, and have a lower availability of healthy foods in their communities [16]. Research has also shown the connections between food insecurity and certain health outcomes. Food insecurity has been associated with higher frailty and odds of falling [17], symptoms of asthma [18], and worsening cognitive functions [19].

### 1.3. Food Environments and Health Outcomes

Having looked at the research on the inequities in food environments for certain population groups, knowing that such food environments also correlate with food insecurity and that food insecurity has been known to correlate with health outcomes, it is imperative that we look at the research on food environments and health outcomes. The most commonly found studies assessing food environments and health outcomes look at BMI or obesity as the health outcome. Research on the topic of access to healthy foods and obesity rates shows that people with greater access to supermarkets had healthier diets and lower rates of BMI and obesity [20,21,22] and those with lesser access to healthy foods or who are in food swamps have higher BMI/obesity rates in general [23,24,25,26,27], in older adults [28], in Black and Hispanic adults and youth [29], and in children, in particular [4]. Results are mixed, with some showing weak correlations and some studies showing higher correlations between food environments and BMI or obesity.

Limited research has also shown associations between the food environment and diabetes. A longitudinal study measuring glycemic control in 14,985 individuals with diabetes found that individuals who had a supermarket within 1600 m of their house had better glycemic control over the course of 3 years [30]. Similar results between areas with more fast-food outlets and convenience stores and higher rates of diabetes have been reported [3,31]. There has also been limited research showing the relationship between food environments and hypertension [32] and cardiovascular issues [33,34,35].

While a considerable amount of research has been carried out on under-resourced neighborhoods and their food environment and, separately, exposure to food swamps and the impacts on BMI/obesity, there is a lack of sufficient research on the food environment and others. This study aims to overcome this gap. We aim to assess the relationship between food environments, specifically food swamps, which denotes an overabundance of fast-food restaurants and convenience stores, and food insecurity on various health outcomes.

## 2. Materials and Methods

### 2.1. Study Area

This study was conducted for the city of Lansing, the capital of Michigan state. Sitting on just under 40 square miles, Lansing has a population of just over 113,400 people [36], 14.8% are 65 years old and over, over 34% are non-white, just under 30% have a Bachelor’s degree or higher, and the median household income is USD 55,366. A total of 22.6% of the population lives below the poverty line, and 19.6% of the households receive food stamps or Supplemental Nutrition Assistance Program (SNAP) assistance [36]. Lansing has its pockets of underserved or vulnerable residents, with some of the higher rates of poverty and minority residents compared to the three counties that it covers parts of (Eaton, Ingham, and Clinton counties), and choosing this city for the study area serves multiple purposes. First, being the capital of the state of Michigan, Lansing has political significance in the state. It also has some major health institutions within its jurisdiction and a transit system (Capital Area Transportation Authority—CATA) that has multiple routes throughout the whole city. Finally, Lansing is adjacent to East Lansing, home of Michigan State University, so the sharing of resident characteristics, entrepreneurship, and economic activity is synergistic.

### 2.2. Data Collection

For this study, we compiled GIS-based data on neighborhood demographic characteristics, health outcomes, and food outlet locations for Lansing, Michigan. The CDC PLACES—Local Data for Better Health dataset provides data for 40 different chronic diseases and other health measurements for the entire U.S. at multiple local area levels, including county, place, census tract, and ZCTA levels. The health-based estimates are based on data from the Behavioral Risk Factor Surveillance System (BRFSS), census decennial population counts and annual county population estimates, and the American Community Survey 5-year estimates [CDC]. We used the 2024 PLACES dataset (the most recent available when this study was conducted) to gather prevalence rates for relevant health outcomes by census tract in Lansing, MI, which are available for download from PLACES by users for secondary analysis. Relevant health outcomes were decided on by determining if they were perceived to possibly have an association with the food environment through the literature reviews conducted. Prevalence rates by census tract for relevant data obtained from CDC PLACES included chronic obstructive pulmonary disease (COPD), coronary heart disease (CHD), depression, diabetes, fair/poor health status, mental distress, physical distress, high blood pressure (BP), high cholesterol, and obesity. Prevalence rates for non-medical social determinants of health were also gathered and included persons 65 and over, no broadband/internet services, crowding, housing cost burden, no high school diploma among adults aged 25 and older, persons below 150% of the poverty level, racial or ethnic minority status, single-parent households, and unemployment. This data was collected from the latest ACS 2023 data that was available at the time of this study. This data was then uploaded to ArcGIS Pro 3.5 to be visually modeled by census tract in the decided study area of Lansing, MI.

Data for the food environment in Lansing, MI, was gathered from ESRI Business Analyst. A list of all food stores, including grocery, wholesale, department, specialty, vitamin, coffee shops, bars, standard sit-down, pizza, and fast-food, was downloaded during September–November of 2024 using the 2024 data published on the website. Each restaurant food location was sorted into one of six categories, including fast-food, ethnic, regular, upscale, niche, and healthy. The categorization of the food environment started with the list of SIC codes for each business in the city. We then kept only the businesses that had codes relating to the food environment, that is, SIC codes beginning with 54 and 58. For each business, we then classified them into groups based on their 4-digit codes and definitions used on the NAICS website. Each business was then further classified based on their 6-digit code classification. All food locations were then uploaded to Arc Pro to be visualized in relation to the various health- and non-health-related social determinants of health. All the data collected was based on the latest information available, and comparison in terms of years was assured. Since we had PLACES data available in 2024, we used the latest census (ACS) data available in 2024 as well to ensure the maximum likelihood of a robust analysis. We have also included maps showing some demographic and health distributions throughout the study area in Appendix A.

### 2.3. Analyses

The unit of analyses was census tracts so that we matched the data from the CDC, which was also available for census tracts. Lansing has 43 tracts, and that is the sample size for this study. With all the data uploaded to Arc Pro, we first standardized the unit of analysis to census tracts by spatially joining (and using “summarize within”) the various geographic levels of data into census tracts to be used for statistical analyses. We used SPSS 27 to carry out the statistical analyses. First, data was treated for skewness, and then we ran a Principal Components Analysis (PCA) to reduce the number of independent variables and address any multicollinearity issues. The treated data also helped with addressing the assumptions for OLS analyses. We then ran regression analyses with the factor loadings for each of the 5 components extracted using varimax rotation. We also ran the regressions using selected variables from each of the 5 components. To address any spatial autocorrelation, we also ran a spatial autoregression analysis in Arc Pro using those health variables that showed clustering of residuals on the Moran’s I test. Lastly, we wanted to see if there were any indirect effects of the food environment on health outcomes, and so we ran a structural equations modeling (SEM) test to check for direct and indirect effects of fast-food restaurants and convenience stores on each of the health outcomes through food insecurity as the mediating variable. The SEM analysis was conducted using the maximum likelihood robust estimator (MLR) and a bootstrap sample of 5000 with replacement.

## 3. Result

The final dataset included 19 independent variables and 10 dependent or health-related variables. The Principal Component Analysis (PCA) resulted in five factors/categories that broadly represented the following themes: income-related, food environment, convenience stores, older adults, and total population, with the last category only having one contributing variable (total population). Table 1 shows the variables in each factor loading and variance explained by each category. Table 2 shows the results of the linear regressions with the PCA categories. What is evident from Table 2 is that the income category is significant for all the health variables, the food environment category is significantly related to physical and mental distress and poor health status, and the convenience category is significantly related to obesity, cholesterol, depression, physical and mental distress, and Poor health status. The last two categories of older adults and total population are only moderately significantly related to CHD and mental distress and depression, respectively.

To make more sense of the regressions, we ran linear regressions with selected variables within each of the five categories identified in the PCA. Within the income category, we selected food insecurity, within the food environment category, the variable selected was the number of fast-food restaurants (FF_Rest.), the variable selected in the convenience category was convenience stores (Conv_Stores), number of older adults (OA) represented the fourth category, while total population (Tot. Pop) was the only variable in the fifth category. Table 3 shows the correlation matrix and collinearity diagnostics for these selected variables. Table 4 shows the results of the regression analyses using these selected variables. Interestingly, this analysis showed that food insecurity was once again significantly related to all health variables, the number of older adults was significantly related to cholesterol, depression, CHD, mental distress, and COPD, while the number of fast-food restaurants, convenience stores, and total population were not significantly related to any of the health outcomes.

We also ran a Moran’s I test on the health outcomes, and those results showed that they were spatially correlated. However, this was expected. There are certain areas in most cities that face the issues of health outcomes being spatially clustered, as those also point to social determinants of health. Additional Moran’s I analyses on the standardized residuals from the OLS regressions showed that obesity, CHD, physical distress, poor health status, and COPD had random distributions, while the residuals for cholesterol, blood pressure, diabetes, depression, and mental distress had clustered distributions. We then ran a spatial autoregression on the five health outcomes mentioned above to account for the spatial autocorrelation. The results of those analyses are presented in Table 5.

Since the selected variables were from each of the PCA components, we expected them to not be correlated, and the VIF statistics show this relationship.

The spatial autoregression (SAR) results are similar to the OLS results. Food insecurity and percentage of older adults are significantly related to many health outcome variables. Surprisingly, the auto-detect model function for SAR reverted to OLS regressions for blood pressure. Since we are testing for significant relationships and not the power of prediction, the original OLS tests seem to suffice, yet the SAR analyses confirmed the associations.

A structural equation model was used to test the hypothesized relationships between Conv_Stores, FF_Rest., FD_Insecurity, and the various health outcomes (see Table 6 for results). The model was estimated using Lavaan 0.6–19 with maximum likelihood estimation. Taking COPD as an example, the standardized path coefficients showed that Conv_Stores had a significant positive effect on FD_Insecurity (β = 20.819, *p* = 0.036), while FF_Rest did not. FD_Insecurity also had a significant positive effect on COPD (β = 0.210, *p* = 0.000). Indirect effects show that convenience stores are indirectly related to COPD via food insecurity as the mediator (β = 0.592, *p* = 0.045), while fast-food restaurants had a direct effect on COPD (β = 0.220, *p* =0.034) and not through a mediator. These results suggest that the number of convenience stores is positively related to food insecurity, and food insecurity mediates the relationship between convenience stores and COPD, while fast-food restaurants had a direct and positive relationship with COPD. Due to the small sample size, maximum likelihood estimation was used to enhance model robustness, and a post hoc power analysis was performed. The model was just-identified (χ^2^(0) = 0.00, *p* = n.a.), indicating a saturated model with no degrees of freedom. Fit indices were therefore trivially perfect (CFI = 1.00, TLI = 1.00, RMSEA = 0.00, SRMR = 0.00), which reflects exact reproduction of the observed covariance matrix rather than empirical model fit. Power analyses results and other coefficients are reported in a table in Appendix B.

## 4. Discussion

The analyses performed in this study show that various socio-demographic characteristics have a statistically significant relationship with health outcomes. When we used PCA to account for multi-collinearity issues, we saw that the income category had a statistically significant relationship with all the health outcome variables used. One notable exception was that cholesterol did not have a significant relationship with any independent variable in the model. The categories of food and convenience stores were statistically and significantly associated with the same set of five of the nine health variables, while OA and total population were only related to two and three of the nine health variables, respectively. An interesting way to look at the health outcome variables is dividing them into perceptions (how you feel: for example, physical distress, mental distress, and poor health status would fall into this category) and diagnosed (a condition that requires a doctor or health professional to diagnose: for example, obesity, BP, diabetes, depression, CHD, and COPD would fall into this category). The perceived group of health outcomes had consistent significant relationships with the income, food, and convenience store categories, while the diagnosed group did not have consistent significant relationships with a specific set of categories.

Since the first set of regression analysis used the PCA categories, it was harder to explain (or understand) the unique contribution of any one variable to the statistical relationships, and hence, we ran linear regressions with select variables from each of the five PCA categories. The results (shown in Table 4) show that food insecurity is the only variable that is consistently significantly (and positively) related to all ten health outcome variables (including cholesterol), followed by older adults being significantly related to nine out of the ten health outcome variables (this category was not related to poor health status). All associations between older adults and health outcomes were positive, except for depression and mental distress, which were negative associations. Conversely, fast-food restaurants and convenience stores were not significantly associated with any of the health outcomes, similar to one study by Rundle et al. [23] yet contrary to many of the studies that have shown that such relationships exist [3,23,24]. Since food insecurity is a complex indicator made up of several variables, it is understandable that it is related to many of the health outcomes. Addressing spatial autocorrelation in the standardized residuals, the SAR analyses on those health variables where the residuals were clustered showed similar results to those of the OLS regression, showing food insecurity and older adults being significantly associated with health outcomes.

Lastly, to ascertain whether the food environment, specifically fast-food restaurants and convenience stores, has an effect on the health status of the resident population, we ran a structural equation modeling test to see if there was a direct or indirect effect of fast-food restaurants and grocery stores on the health outcomes via food insecurity as the mediating variable.

The results shown in Table 6 indicate a couple of consistent trends. Convenience stores have no direct relationship with any of the health outcomes, yet they have an indirect relationship with eight of the ten health outcomes via food insecurity (it is not related to cholesterol and CHD). On the contrary, fast-food outlets have a direct relationship with five of the ten health outcomes (diabetes, CHD, physical distress, poor health status, and COPD) but no indirect effect on any health outcome via food insecurity. A plausible explanation for this is that people going to fast-food outlets are directly consuming unhealthy foods that would affect their health, while those going to convenience stores might be looking to purchase goods for immediate use (like over-the-counter medicines) that might not directly be consumed or affect their health. This set of analyses shows the negative impacts of food swamps on health outcomes, either directly or indirectly.

Policy implications from this study point towards placing greater importance on curbing fast-food outlets in areas with fewer options for healthy, fresh, nutritious, and affordable food. This has a direct impact on resident health. An assessment of areas where there are underserved residents with or at risk of food insecurity would denote areas for policy implementation encouraging creative ways to include outlets with healthy, affordable, and nutritious food. Secondly, convenience stores should be allowed to locate in areas after careful consideration of characteristics pointing towards residents that could face food insecurity. Policies encouraging convenience stores to stock healthy foods would also go a long way in providing access to such foods for those residents for whom such convenience stores are in close proximity. We do not aim to dictate where fast-food outlets and convenience stores locate precisely; rather, we urge municipalities to balance out the food environment with healthy and affordable food options so that residents have the option to improve their dietary intake and health outcomes. Underserved population groups might not have the choice to improve their health through healthy food consumption if they live in food swamps.

This study has its own limitations in that the data is secondary (on health outcomes) and there is a need to acknowledge the inherent limitations of using the PLACES dataset, especially for tracts with low population levels. Also, the study would benefit from actual data on visit frequencies to the food environments, especially fast-food restaurants and convenience stores. Knowing whether people living in food swamps actually visit these places (and the frequency of such visits/purchases) would prove a stronger connector between food environments and health outcomes. The small sample size (*n* = 43) denoting the number of census tracts in the city may influence the analyses’ results. However, we could not effectively address this issue as it is an objective (number of tracts in the city) rather than a subjective or controllable attribute of the study. While we do not have an explanation for why cholesterol was the only health outcome that was not directly or indirectly related to food environments, this is certainly a connection that should be explored further in future studies.

## 5. Conclusions

The results from this study point to the importance of social determinants of health, which essentially posit that the environment in which we live, work, and play influences our health, and the food environment is one of the key determinants of this environment. Access and proximity to quality, affordable, and nutritious food affects our health, especially for those that are disadvantaged. Although this study wanted to stress objectively on the food environment and the health outcomes of residents of such environments, we cannot effectively rule out the inequities of such environments and health outcomes for disadvantaged population groups. In fact, this study shows just that! Food insecurity is faced disproportionately by disadvantaged groups of people, and their living and food environments are negatively affecting their health.

## Figures and Tables

**Table 1:** Results from the Principal Components Analysis (PCA).

	Income	Food	Convenience Stores	Older Adults	Total Population
% of Variance Explained	40.912	19.921	8.833	5.849	5.270
Variables within each component (ordered by their contributing roles and factor loadings shown in parentheses)	Food insecurity (0.952) On food stamps (0.949) No transportation (0.942) Housing insecurity (0.942) No insurance (0.913) Poverty (0.820) Housing cost burden (0.773) Percent minority (0.739) High school degree (0.736)	All restaurants (0.945) Ethical restaurants (0.910) Fast-food restaurants (0.842) All grocery stores (0.808)	Unemployment (0.825) Convenience stores (0.771)	No Internet (0.638) Older adults (0.609)	Total Population (0.969)

**Table 2:** Linear regression results with PCA categories.

Dependent Variables	Obesity	Cholesterol	BP	Diabetes	Depression	CHD	Physical Distress	Mental Distress	Poor Health	COPD
Income t value	0.859 *** 11.962		0.541 *** 4.080	0.713 *** 6.381	0.573 *** 4.911	0.400 *** 2.877	0.848 *** 10.908	0.835 *** 12.454	0.903 *** 15.310	0.731 *** 6.917
Food t value	0.133 * 1.852		−0.004 −0.031	0.140 1.248	0.222 * 1.904	0.121 0.868	0.158 ** 2.028	0.263 *** 3.930	0.191 *** 3.236	0.150 1.418
Conv_Stores t value	0.193 ** 2.692		0.075 0.569	0.026 0.234	0.271 ** 2.327	−0.008 −0.056	0.169 ** 20.177	0.208 *** 3.107	0.135 ** 2.293	0.144 1.360
OA t value	0.018 0.245		0.233 * 1.758	0.127 1.135	−0.056 −0.483	0.347 ** 2.500	0.052 0.670	−0.076 −1.135	0.012 0.212	0.136 1.289
Tot0. Pop0. t value	−0.143 * −1.997		0.121 0.913	0.069 0.621	−0.238 ** −2.039	0.097 0.700	−0.082 −1.057	−0.149 ** −2.226	−0.064 −1.077	−0.051 −0.487
R^2^	0.788		0.279	0.488	0.442	0.208	0.752	0.816	0.857	0.542

Table is reporting standardized coefficients. * = *p* < 0.1, ** = *p* < 0.05, *** = *p* < 0.01; *n* = 42; shaded column shows model not significant.

**Table 3:** Correlation matrix and VIF statistics for selected variables in each of the PCA categories.

	Food Insecurity	Fast-Food Restaurants	Convenience Stores	Older Adults	Total Population
Food Insecurity	1				
Fast-Food Restaurants	0.2045	1			
Convenience Stores	0.3275	0.2701	1		
Older Adults	−0.5192	0.1086	−0.2025	1	
Total Population	−0.1498	0.1152	0.0412	0.3049	1
VIF	1.571	1.189	1.200	1.580	1.121

**Table 4:** Linear regression results with selected variables in each of the PCA categories.

Dependent Variables	Obesity	Cholesterol	BP	Diabetes	Depression	CHD	Phy. Distress	Mtl. Distress	Poor Health	COPD
Food_Insecurity t value	0.910 *** 8.471	0.331 ** 2.148	0.830 *** 6.045	0.938 *** 7.716	0.301 ** 2.165	0.740 *** 5.153	0.947 *** 9.612	0.564 *** 5.794	0.968 *** 13.121	0.897 *** 7.214
FF_Rest t value	−0.012 −0.124	−0.185 −10.381	−0.111 −0.926	0.027 0.251	0.135 1.129	0.074 0.590	0.036 0.423	0.139 1.642	0.070 10.093	0.051 0.474
Conv_Stores t value	0.089 0.943	0.090 0.668	0.074 0.619	−0.052 −0.492	0.079 0.657	−0.034 −0.272	0.027 0.317	−0.015 −0.173	−0.018 −0.279	0.018 0.169
OA t value	0.200 * 1.854	0.771 *** 4.993	0.636 *** 4.615	0.437 *** 3.582	−0.408 *** −2.951	0.636 *** 4.413	0.195 * 1.971	−0.372 *** −3.815	0.087 1.182	0.355 *** 2.845
Tot. Pop. t value	−0.006 −0.065	0.080 0.614	0.119 1.028	0.128 1.242	−0.208 * −1.786	0.078 0.641	0.032 0.380	−0.141 * −1.719	0.039 0.628	0.052 0.498
Adj. R^2^	0.692	0.366	0.496	0.605	0.493	0.448	0.741	0.747	0.855	0.586

Table is reporting standardized coefficients. * = *p* < 0.1, ** = *p* < 0.05, *** = *p* < 0.01; *n* = 42.

**Table 5:** Spatial autoregression results.

	Cholesterol	BP	Diabetes	Depression	Mental Distress
Food Insecurity	0.186 **	0.616 ***	0.274 ***	0.100	0.274 ***
FF_Rest	−0.105	−0.182	0.067	0.063	0.144 **
Conv_Stores	0.830	0.700	−0.032	0.205	−0.124
OA	0.476 ***	0.592 ***	0.174 ***	−0.128 **	−0.205 ***
Tot. Pop	0.000	0.000	0.000	−0.000	−0.000
Model	LAG	OLS	LAG	LAG	LAG
Lag Y (rho)	0.723 ***	n/a	0.649 **	0.361	0.196
Spatial Pseudo R^2^	0.460	n/a	0.523	0.585	0.782
A-K Test	Not significant	n/a	Not significant	Not significant	Not significant

** = *p* < 0.05, *** = *p* < 0.01.

**Table 6:** Results of the SEM analyses.

Dependent Variable	Convenience Stores	Fast-Food Restaurants	Convenience Stores	Fast-Food Restaurants	R^2^
	Direct Effects (β)		Indirect Effect (via Food Insecurity as the Mediator) (β)		
Obesity	0.488	0.065	10.571 **	0.231	0.702
Cholesterol	0.233	0.194	−0.181	−0.027	0.022
BP	0.349	0.257	0.788 *	0.116	0.162
Diabetes	−0.110	0.168 **	0.602 **	0.089	0.379
Depression	0.406	−0.044	0.550 *	0.187	0.374
CHD	−0.014	0.109 ***	0.153	0.023	0.156
Physical Distress	0.064	0.102 *	0.957 **	0.141	0.746
Mental Distress	0.096	−0.026	10.155 **	0.170	0.643
Poor Health	−0.125	0.191 *	10.977 **	0.291	0.866
COPD	−0.013	0.133 **	0.592 **	0.087	0.553

* = *p* < 0.1, ** = *p* < 0.05, *** = *p* < 0.01; *n* = 42.

**Figure A1:**
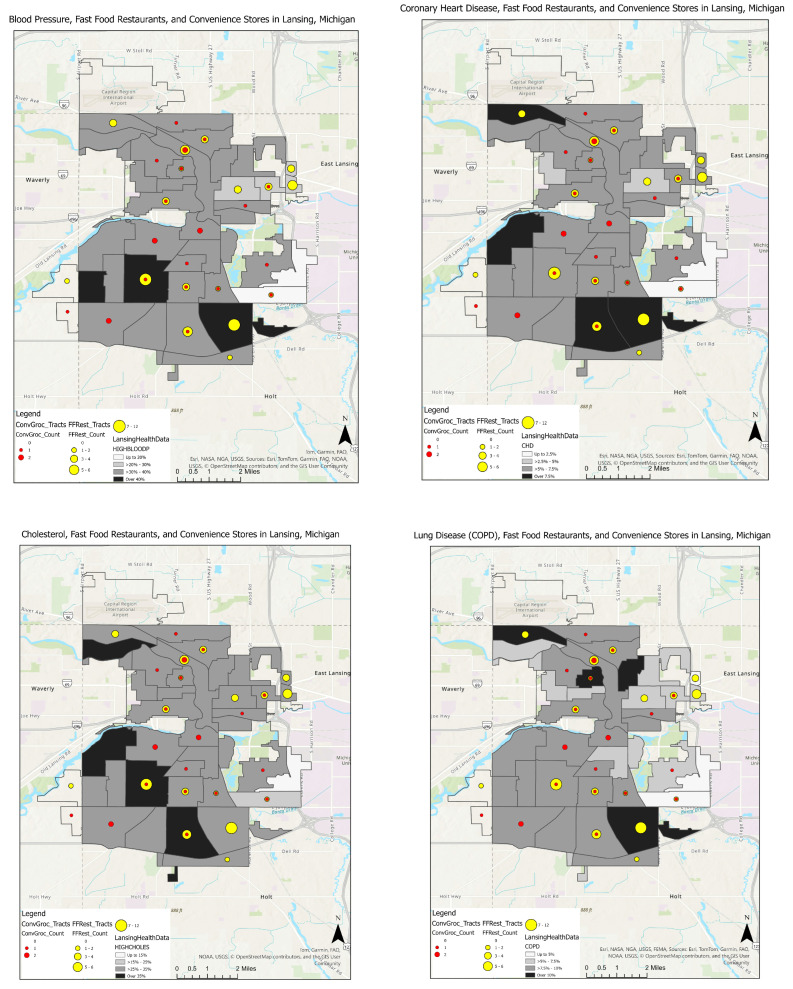

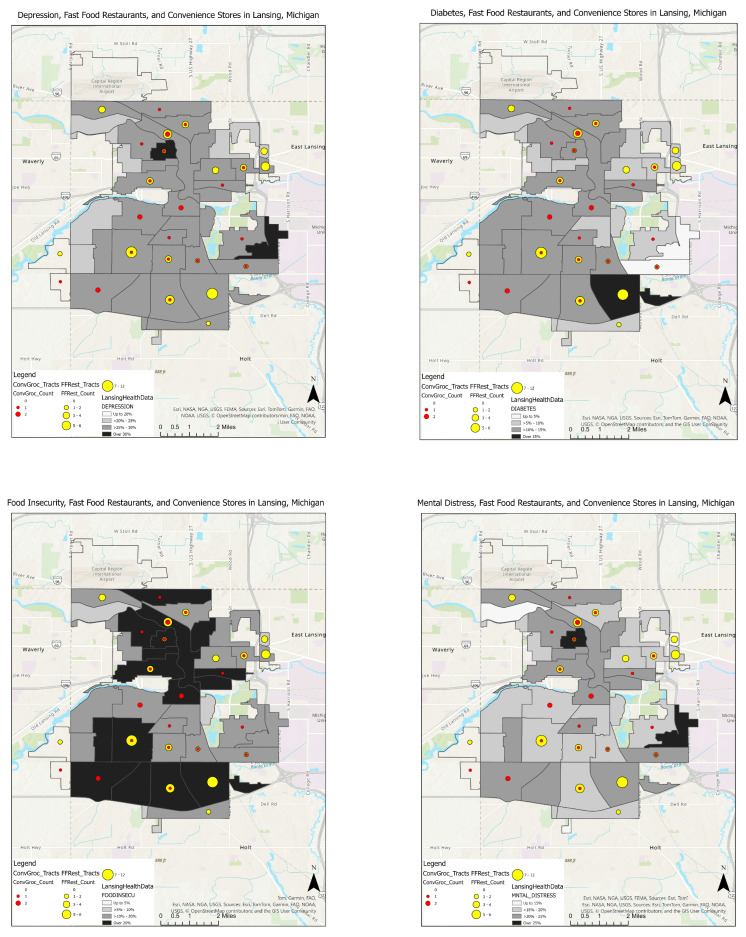

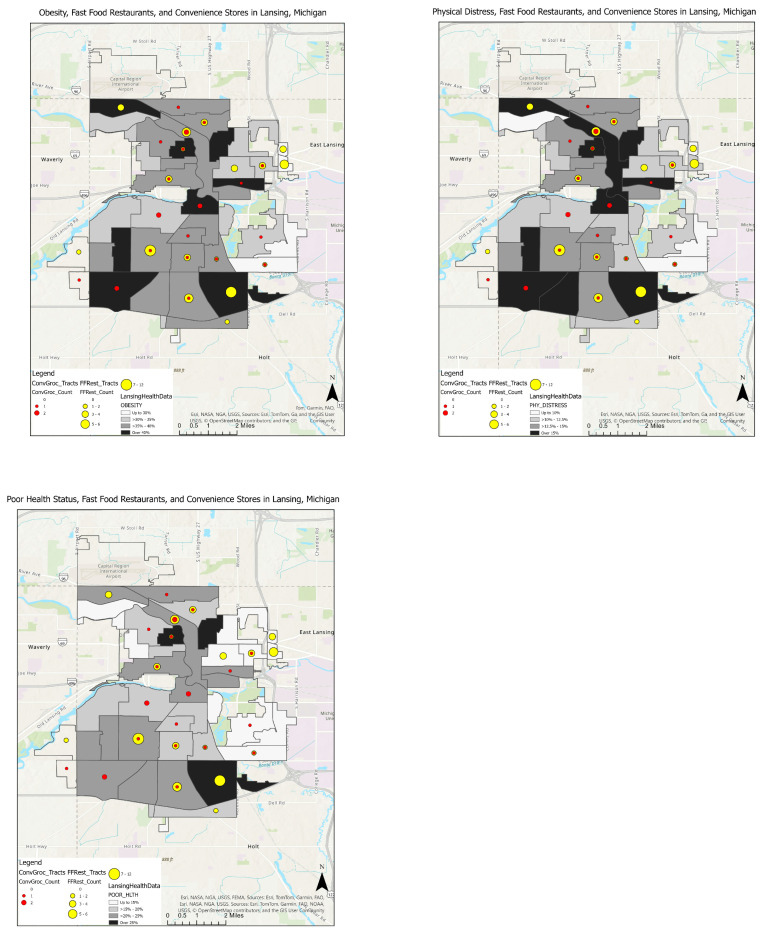
Maps showing spatial distribution of health variables.

## Data Availability

The data presented in this study are derived from the following resources available in the public domain: (CDC PLACES) https://www.cdc.gov/places/tools/data-portal.html Accessed on 15 September 2024; (U.S. Census) https://www.socialexplorer.com/home/dataset-entry/us-census-data Accessed on 15 September 2024.

## Appendix B

**Table A1:** Model indicators for the SEM analyses.

Dependent Variable	Power Analysis: a1	Power Analysis: b	Power Analysis: % Explained	Std. Error (CI Lower—CI Upper)
Obesity: Fast food direct effect Conv store indirect effect	0.291	0.801	0.428	0.094 (−0.120–0.249) 10.342 (0.188–50.450)
Cholesterol: Fast food direct effect Conv store indirect effect	0.291	−0.100	0.011	0.153 (−0.106–0.494) 10.342 (0.188–50.450)
BP Fast food direct effect Conv store indirect effect	0.291	0.333	0.129	0.216 (−0.167–0.680) 10.342 (0.188–50.450)
Diabetes: Fast food direct effect Conv store indirect effect	0.291	0.555	0.326	0.078 (0.015–0.322) 10.342 (0.188–50.450)
Depression: Fast food direct effect Conv store indirect effect	0.291	0.573	0.337	0.070 (−0.181–0.093) 10.342 (0.188–50.450)
CHD: Fast food direct effect Conv store indirect effect	0.291	0.272	0.079	0.041 (0.029–0.189) 10.342 (0.188–50.450)
Physical distress: Fast food direct effect Conv store indirect effect	0.291	0.827	0.434	0.059 (−0.014–0.219) 10.342 (0.188–50.450)
Mental distress: Fast food direct effect Conv store indirect effect	0.291	0.801	0.428	0.075 (−0.173–0.120) 10.342 (0.188–50.450)
Poor health status: Fast food direct effect Conv store indirect effect	0.291	0.905	0.452	0.101 (−0.007–0.390) 10.342 (0.188–50.450)
COPD: Fast food direct effect Conv store indirect effect	0.291	0.680	0.397	0.059 (0.018–0.248) 10.342 (0.188–50.450)

## References

[1] Center for Disease Control About Chronic Diseases. https://www.cdc.gov/chronic-disease/about/index.html.

[2] Boone-Heinonen J., Gordon-Larsen P., Kiefe C., Shikany J., Lewis C., Popkin B.M. (2011). Fast Food restaurants and food stores longitudinal associations with diet in young to middle-aged adults: The CARDIA study. Arch. Intern. Med..

[3] Ahern M., Brown C., Dukas S. (2011). A national study of the association between food environments and county-level health outcomes. J. Rural Health.

[4] Galvez M., Hong L., Choi E., Liao L., Godbold J., Brenner B. (2009). Childhood obesity and neighborhood food-store availability in an inner-city community. Acad. Pediatr..

[5] Block J.P., Scribner R.A., DeSalvo K.B. (2004). Fast-food, race/ethnicity, and income: A geographic analysis. Am. J. Prev. Med..

[6] Powell L.M., Slater S., Mirtcheva D., Bao Y., Chaloupka F.J. (2006). Food store availability and neighborhood characteristics in the United States. Prev. Med..

[7] Holston D., Cater M., Tuuri G., O’Neil C. (2019). Exploring the rural nutrition environment: A case study from Louisiana. Curr. Dev. Nutr..

[8] Bower K.M., Thorpe R., Rohde C., Gaskin D.J. (2014). The intersection of neighborhood racial segregation, poverty, and urbanicity and its impact on food store availability in the United States. Prev. Med..

[9] Smoyer-Tomic K., Spence J., Raine K., Amrhein C., Cameron N., Yasenovskiy V., Cutumisu N., Hemphill E., Healy J. (2008). The association between neighborhood socioeconomic status and exposure to supermarkets and fast food outlets. Health Place.

[10] LeDoux T., Vojnovic I. (2014). Examining the role between the residential neighborhood food environment and diet among low-income households in Detroit, Michigan. Appl. Geogr..

[11] Burns C.M., Inglis A.D. (2007). Measuring food access in Melbourne: Access to healthy and fast foods by car, bus and foot in an urban municipality in Melbourne. Health Place.

[12] Gordon C., Purciel-Hill M., Ghai N.R., Kaufman L., Graham R., Van Wye G. (2011). Measuring food deserts in New York City’s low-income neighborhoods. Health Place.

[13] Sloane D., Diamant A., Lewis L., Yancey A., Flynn G., Nascimento L., McCarthy W., Guinyard J., Cousineau M. (2003). Improving the nutritional resource environment for healthy living through community-based participatory research. J. Gen. Intern. Med..

[14] Zenk S., Lachance L., Schultz A., Mentz G., Kannan S., Ridella W. (2009). Neighborhood retail food environment and fruit and vegetable intake in multiethnic urban population. Am. J. Health Promot..

[15] Gundersen C., Ziliak J.P. (2015). Food Insecurity And Health Outcomes. Health Aff..

[16] Larson N., Laska M., Neumark-Sztainer D. (2020). Food insecurity, diet quality, home food availability, and health risk behaviors among emerging adults: Findings from the EAT 2010–2018 study. Am. J. Public Health.

[17] Wennberg A.M., Ek S., Na M. (2024). Food insecurity, vision impairment, and longitudinal risk of frailty and falls in The National Health and Aging Trends Study. J. Frailty Aging.

[18] de Cássia Ribeiro-Silva R., Oliveira-Assis A.M., Junqueira S.B., Fiaccone R.L., Dos Santos S.M.C., Barreto M.L., de Jesus Pinto E., da Silva L.A., Rodrigues L.C., Alcantara-Neves N.M. (2014). Food and nutrition insecurity: A marker of vulnerability to asthma symptoms. Public Health Nutr..

[19] Wang H., El-Abbadi N. (2024). Food insecurity, race and ethnicity, and cognitive function among United States older adults. J. Nutr..

[20] Dubowitz T., Ghosh-Dastidar M., Eibner C., Slaughter M., Fernandes M., Whitsel E., Bird C., Jewell A., Margolis K., Li W. (2012). The Women’s Health Initiative: The food environment, neighborhood socioeconomic status, BMI, and blood pressure. Obesity.

[21] Larson N.I., Story M.T., Nelson M.C. (2009). Neighborhood Environments Disparities in Access to Healthy Foods in the US. Am. J. Prev. Med..

[22] Rundle A., Neckerman K., Freeman L., Lovasi G., Purciel M., Quinn J., Richards C., Sircar N., Weiss C. (2009). Neighborhood food environment and walkability predict obesity and New York city. Environ. Health Persp..

[23] Bodor J., Rice J., Farley T., Swalm C., Rose D. (2010). The association between obesity and urban food environments. J. Urban Health.

[24] Burgoine T., Forouhi N., Griffin S., Wareham N., Monsivais P. (2014). Associations between exposure to takeaway food outlets, takeaway food consumption, and body weight in Cambridgeshire, UK: Population based, cross sectional study. BMJ Brit. Med. J..

[25] Burgoine T., Sarkar C., Webster C., Monsivais P. (2018). Examining the interaction of fast-food outlet exposure and income on diet and obesity: Evidence from 51,361 UK Biobank participants. Int. J. Behav. Nutr. Phys. Act..

[26] Cooksey Stowers K., Jiang Q., Atoloye A., Lucan S., Gans K. (2020). Racial differences in perceived food swamp and food desert exposure and disparities in self-reported dietary habits. Int. J. Environ. Res. Public Health.

[27] Murphy M., Badland H., Jordan H., Koohsari M., Giles-Corti B. (2018). Local food environments, suburban development, and BMI: A mixed methods study. Int. J. Environ. Res. Public Health.

[28] Li F., Harmer P., Cardinal B., Bosworth M., Johnson-Shelton D. (2009). Obesity and the built environment: Does the density of neighborhood fast-food outlets matter?. Am. J. Health Promot..

[29] Kraft A.N., Thatcher E.J., Zenk S.N. (2020). Neighborhood food environment and health outcomes in U.S. low-socioeconomic status, racial/ethnic minority, and rural populations: A systematic review. J. Health Care Poor Underserved.

[30] Rosenberg D.E., Cruz M.F., Mooney S.J., Bobb J.F., Drewnowski A., Moudon A.V., Cook A.J., Hurvitz P.M., Lozano P., Anau J. (2024). Neighborhood built and food environment in relation to glycemic control in people with type 2 diabetes in the Moving to Health Study. Health Place.

[31] Phillips A., Rodriguez H. (2019). Adults with diabetes residing in “food swamps” have higher hospitalization rates. Health Serv. Res..

[32] Mujahid M., Roux A., Morenoff J., Raghunathan T., Cooper R., Ni H., Shea S. (2008). Neighborhood characteristics and hypertension. Epidemiology.

[33] Landaeta-Diaz L., Vergara-Perucich F., Aguirre-Nunez C., Cancino-Contrearas F., Correa-Parra J., Ulloa-Leon F. (2024). Urban food deserts and cardiovascular health: Evaluating the impact of nutritional inequities on elderly populations in Santiago. Appl. Sci..

[34] Ly C., Essman M., Zimmer C., Ng S.W. (2020). Developing an index to estimate the association between the food environment and CVD mortality rates. Health Place.

[35] Mooney S.J., Lemaitre R.N., Siscovick D.S., Hurvitz P., Goh C.E., Kaufman T.K., Zulaika G., Sheehan D.M., Sotoodehnia N., Lovasi G.S. (2018). Neighborhood food environment, dietary fatty acid biomarkers, and cardiac arrest risk. Health Place.

[36] U.S. Census Bureau, U.S. Department of Commerce 2024 ACS Demographic and Housing Estimates. https://www.socialexplorer.com/explore-tables.

